# Investigation of Cross-Linked Chitosan-Based Membranes as Potential Adsorbents for the Removal of Cu^2+^ Ions from Aqueous Solutions

**DOI:** 10.3390/ma16051926

**Published:** 2023-02-25

**Authors:** Irene Vlachou, Georgios Bokias

**Affiliations:** Department of Chemistry, University of Patras, GR-26504 Patras, Greece

**Keywords:** chitosan, copolymers, glycidyl methacrylate, chemical cross-linking, polymeric membranes, copper sulfate, adsorption capacity

## Abstract

Rapid industrialization has led to huge amounts of organic pollutants and toxic heavy metals into aquatic environment. Among the different strategies explored, adsorption remains until the most convenient process for water remediation. In the present work, novel cross-linked chitosan-based membranes were elaborated as potential adsorbents of Cu^2+^ ions, using as cross-linking agent a random water-soluble copolymer P(DMAM-co-GMA) of glycidyl methacrylate (GMA) and N,N-dimethylacrylamide (DMAM). Cross-linked polymeric membranes were prepared through casting aqueous solutions of mixtures of P(DMAM-co-GMA) and chitosan hydrochloride, followed by thermal treatment at 120 °C. After deprotonation, the membranes were further explored as potential adsorbents of Cu^2+^ ions from aqueous CuSO_4_ solution. The successful complexation of copper ions with unprotonated chitosan was verified visually through the color change of the membranes and quantified through UV-vis spectroscopy. Cross-linked membranes based on unprotonated chitosan adsorb Cu^2+^ ions efficiently and decrease the concentration of Cu^2+^ ions in water to a few ppm. In addition, they can act as simple visual sensors for the detection of Cu^2+^ ions at low concentrations (~0.2 mM). The adsorption kinetics were well-described by a pseudo-second order and intraparticle diffusion model, while the adsorption isotherms followed the Langmuir model, revealing maximum adsorption capacities in the range of 66–130 mg/g. Finally, it was shown that the membranes can be effectively regenerated using aqueous H_2_SO_4_ solution and reused.

## 1. Introduction

The biopolymer family mainly consists of polyesters, proteins, lipids, and polysaccharides. The most utilized carbonaceous materials worldwide of the latter category, owing to their natural abundance and chemical reactivity, are cellulose and chitin [1,2]. Chitin is a linear aminopolysaccharide with high crystallization ability and poor solubility in water. By chemical or biological hydrolysis of chitin, deacetylation takes place. Usually, when the degree of deacetylation, DD, is higher than 60%, the product is called chitosan. Chitosan mainly consists of D-glucosamine, and due to the partial deacetylation of chitin also contains N-acetyl-D-glucosamine (Figure S1). Its physicochemical properties, such as solubility, are strongly dependent on DD and other factors, such as acidity, molar mass, temperature, and ionic strength [1,2]. It is widely used in the pharmaceutical and biomedicine industries, due to biological properties such as antimicrobial/antifungal action and biocompatibility [3,4,5,6,7]. Moreover, chitosan finds applications in several technological fields [8,9,10,11,12,13,14], e.g., in the food and textile industries.

Concerning technological applications, the contribution of chitosan-based materials in quality environmental assurance has attracted intensive research interest. Specifically, in the case of air pollution, a characteristic is the utilization of chitosan-based fibers as antibacterial air filters with highly efficient moisture resistance [15]. Moreover, numerous works are related to the use of chitosan in the field of water purification for the adsorption of dyes [16,17,18,19,20] and heavy metals, such as Cu(II), Ni(II), Pb(II), Cd(II), Zn(II), Co(II), Hg(II), Cr(VI), As(V), U(VI) and other rare earth elements [16,17,19,20,21,22]. Water purification through adsorption is often preferable compared to other highly effective techniques, such as degradation and chemical precipitation, due to the low cost of adsorbent materials and speedy results. As far as adsorption of metal ions is concerned, intramolecular and intermolecular chelation between the ions and functional groups of chitosan (mostly amine groups, and possibly hydroxyl groups) takes place (Figure S2). For large-scale applications, chitosan is used as powder, fibers, flakes, nanoparticles and membranes. Chemical cross-linking is often applied, and chitosan-based materials with higher mechanical stability are obtained through the reaction with reagents containing functional groups, such as carboxylic groups, aldehydes, epoxides or others [17].

**Figure 1:**
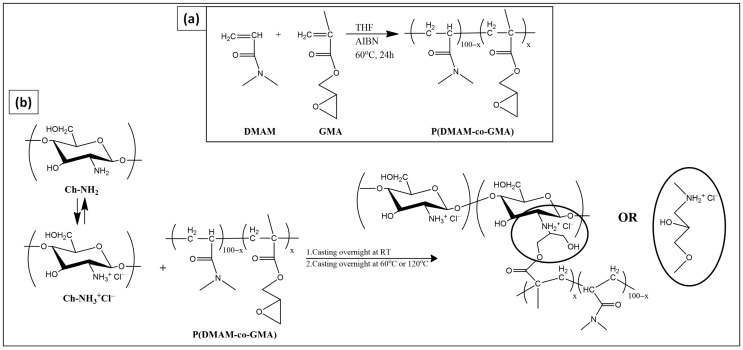
Schematic representation of the chemical synthesis of (**a**) P(DMAM-co-GMA) copolymer and (**b**) cross-linked chitosan-based membranes.

Aiming at novel biocidal materials, in our laboratory, we have developed a methodology for the preparation of cross-linked membranes and coatings through the reaction of glycidyl methacrylate (GMA)-containing copolymers with supplementary polymers containing functional groups such as carboxylic or amine groups [23,24,25,26]. In the present work, this methodology is extended, and we explore the reaction of such GMA-containing copolymers with chitosan. Thus, a water-soluble copolymer of GMA and N,N-dimethylacrylamide (DMAM), P(DMAM-co-GMA) was synthesized and mixed in aqueous solution with chitosan hydrochloride (Ch-NH_3_^+^Cl^−^) to obtain cross-linked chitosan membranes through solution casting and subsequent thermal treatment at high temperature (Figure 1). The potential application of these membranes as adsorbents for metal ions, such as Cu^2+^ ions from aqueous CuSO_4_ solutions, was investigated after membrane deprotonation through immersion in aqueous alkaline solution.

## 2. Materials and Methods

Materials: Chitosan (Ch-NH_2_) (DD > 75% and MW = 310–375 kDa), the monomers N,N-dimethylacrylamide (DMAΜ) and glycidyl methacrylate (GMA), the initiator azobisisobutyronitrile (AΙΒΝ) as well as sulfuric acid (H_2_SO_4_) 99%*w/w* and the solvents N,N-dimethylformamide (DMF), tetrahydrofuran (THF), dimethylacetamide (DMA) and hexane were purchased from Sigma Aldrich. The solvents dimethyl sulfoxide (DMSO), chloroform (CHCl_3_), ethanol and methanol were purchased from Fischer. Anhydrous copper sulphate (CuSO_4_) powder was purchased from Merck, while sodium chloride (NaCl) and micropearls of sodium hydroxide (NaOH) were purchased from Honeywell and Lach-Ner, respectively. Ultrapure water was obtained by means of an SG water purification unit.

Preparation of chitosan hydrochloride, Ch-NH_3_^+^Cl^−^: Firstly, 10 g of chitosan (Ch-NH_2_) was added to a solution of HCl 1 M (150 mL) and the mixture was magnetically stirred at room temperature for 24 h. The initial turbid/phase-separated mixture turned gradually to transparent when purified through dialysis. The product was finally recovered through freeze-drying.

Synthesis of P(DMAM-co-GMA) copolymer: A P(DMAM-co-GMA) copolymer with a molar feed composition of 30% (mol/mol) was synthesized through free radical polymerization of 6.11 g (43 mmol) GMA and 10 g (100 mmol) DMAM, dissolved in 55 mL THF (total monomer concentration 5%*w/v*). The solution was deaerated and 16.5 mg (1 mmol) of the initiator AIBN (0.1% mol/mol over the total monomer concentration) was added. The reaction was left to proceed under magnetic stirring at 65 °C under Ν_2_ for 24 h. The final product was precipitated in hexane, washed with hexane and dried at 40 °C. For reasons of comparison, the two homopolymers, namely poly(N.N-dimethylacrylamide) (PDMAM) and poly(glycidyl methacrylate) (PGMA), were similarly synthesized.

Preparation of cross-linked chitosan-based membranes with P(DMAM-co-GMA): As an example, for the preparation of the membrane Ch-NH_3_^+^Cl^−^/P(DMAM-co-GMA) 7/3 with a feed mass content 70% (*w/w*) of protonated chitosan, 0.21 g Ch-NH_3_^+^Cl^−^ and 0.09 g P(DMAM-co-GMA) were dissolved in 4.2 mL and 1.8 mL water, respectively. After complete dissolution, the two solutions were mixed and magnetically stirred at room temperature for 30 min. The cross-linked membrane was obtained through casting at 60 °C or 120 °C for 24 h. The membranes Ch-NH_3_^+^Cl^−^/P(DMAM-co-GMA) 8/2, Ch-NH_3_^+^Cl^−^/P(DMAM-co-GMA) 9/1 and Ch-NH_3_^+^Cl^−^/P(DMAM-co-GMA) 5/5 were also prepared following a similar procedure.

The unprotonated cross-linked chitosan-based membranes, Ch-NH_2_/P(DMAM-co-GMA), were prepared after immersion in aqueous NaOH 0.1 M solution at room temperature for 24 h without agitation. The final membranes were purified with ultrapure water and dried at 80 °C.

Adsorption studies: The adsorption capacity of cross-linked chitosan-based membranes was studied through batch experiments. Specifically, a piece of Ch-NH_2_/P(DMAM-co-GMA) membrane (m = 5–50 mg) was immersed in 5 mL of aqueous solution of copper sulfate (concentration range 0.1 mM–20 mM) at room temperature with or without magnetic stirring for various immersion times (t = 0–2 days).

The color of the membranes gradually turned to blue. The water uptake of the membranes was determined gravimetrically after removal from the solution and drying at 80 °C, while the concentration of CuSO_4_ solution was determined through UV-vis spectroscopy.

Desorption Studies: The regeneration of the Ch-NH_2_/P(DMAM-co-GMA) membrane was carried out through the immersion of the membrane in aqueous H_2_SO_4_ solution c = 2.5 mM. The successful desorption of copper ions was perceivable from the change in membrane color (blue to brown) and was confirmed by UV-vis spectroscopy. The adsorption–desorption study was repeated for three cycles.

Characterization Techniques: The samples were characterized through attenuated total reflectance—Fourier-transform infrared spectroscopy (ATR-FTIR), Bruker Optics GmbH, in the wavenumber range 300–3800 cm^−1^ and nuclear magnetic resonance (^1^H NMR) using D_2_O and CDCl_3_ as solvent. Molecular weight and polydispersity of the copolymer were determined through size-exclusion chromatography (SEC). For scanning electron microscopy (SEM), a JSM-6300 Jeol scanning microscope was used, in combination with energy-dispersive X-ray spectroscopy (EDS). Finally, UV-vis spectra were recorded on a Hitachi UV 1800 UV-vis spectrophotometer in the 190–1100 nm range at 25 °C, equipped with quartz cell with an optical path length of 1 cm.

Method of Bathocuproine: This method is based on bathocuproine, a reagent that forms complexes with Cu^+^, exhibiting a strong absorbance at 400–600 nm. Hydroxylamine is used as reducing agent of copper ions (Cu^2+^ to Cu^+^). In this work, absorbance at 480 nm was measured by LCK 329 copper of HACH LANGE GMBH.

Determination of Water Uptake and Soluble Content: A sample (10 mg) of the cross-linked chitosan-based membranes was immersed in pure water for 24 h at room temperature without agitation. Water uptake was determined by the equation:

(1) $$\mathrm{Water}\;\mathrm{Uptake}\%=\frac{W_{\mathrm{wet}}-W_{o}}{W_{o}}\%$$

where W_o_ (mg) is the initial mass of the membrane and W_wet_ (mg) is the final mass of the membrane after immersion in water.

The soluble content was calculated after drying the swelled samples at 80 °C for 24 h by the following equation:

(2) $$\mathrm{Soluble}\;\mathrm{Content}\%=\frac{W_{o}-W_{\mathrm{dry}}}{W_{o}}\%$$

where W_o_ (mg) is the initial mass of the membrane before immersion in the aqueous solution and W_dry_ (mg) is the mass of the membrane after immersion and drying at 80 °C.

## 3. Results and Discussion

### 3.1. Synthesis and Characterization of Cross-Linked Chitosan-Based Membranes

For the preparation of the membranes through solvent casting, soluble chitosan derivatives are a prerequisite. As expected [1], the solubility of chitosan was insignificant after immersion in organic solvents (DMA, THF, DMF, DMSO, CHCl_3_) and H_2_O, at room or high temperature, owing to the high level of crystallinity of this biopolymer. The solubility limitation can be overcome through modification of its functional groups or protonation of amine groups by a strong inorganic acid (e.g., HCl) or organic acid (e.g., formic acid) to attain a pH value lower than pKa. In this work, the solubility of chitosan was achieved through protonation, namely the preparation of the hydrochloric derivative, Ch-NH_3_^+^Cl^−^, as depicted in Figure S3. This derivative is soluble in pure water and low-concentration salt solutions. In fact, from the ^1^H NMR spectrum in D_2_O (Figure S4) both D-glucosamine and N-acetyl-D-glucosamine units were identified and the deacetylation degree, DD, of the product was determined to be 85%.

For the chemical cross-linking, the reaction of the amine groups of chitosan with the epoxide ring of glycidyl methacrylate (GMA) was explored (Figure 1b). Since GMA (and the respective homopolymer) is not water-soluble, copolymerization of GMA with the highly hydrophilic monomer N,N-dimethylacrylamide (DMAM) was performed, as depicted in Figure 1a. To ensure water-solubility, a feed composition with a relatively low GMA content, namely 30% mol/mol, was chosen. The copolymer was characterized through ATR-FTIR and ^1^H NMR spectroscopy. The number average, M_n_, weight average, and M_w_, molar mass, of the copolymer were found M_w_ = 8600 and M_n_ = 4600, respectively, through size-exclusion chromatography (SEC).

**Figure 2:**
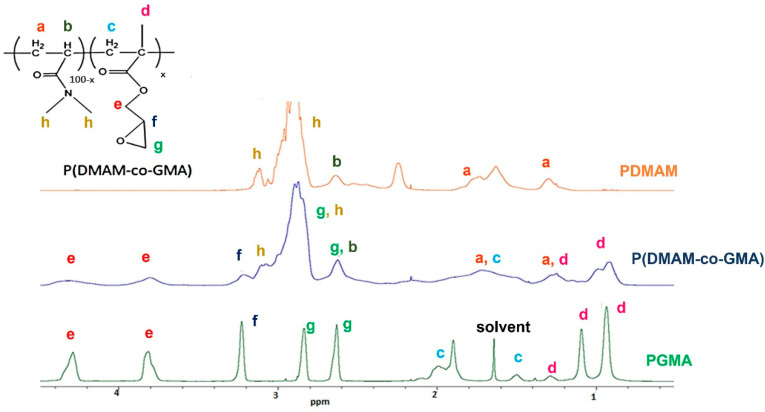
^1^H NMR spectrum of the copolymer P(DMAM-co-GMA) in CDCl_3_ in comparison with the spectra of the two homopolymers, PDMAM and PGMA.

Figure 2 presents the ^1^H NMR spectrum of P(DMAΜ-co-GMA) copolymer in CDCl_3_ in comparison with the spectra of the two homopolymers PDMAM and PGMA. Based on the ^1^H NMR structural analysis, the -CH_2_- groups (e) of GMA appear at 3.8 ppm and 4.3 ppm, while the -CH- group (f) is observed at around 3.2 ppm. The asymmetric double peak (h) observed in the range 3.1–2.8 ppm in the spectrum of PDMAM is characteristic of the different stereochemical configurations of -CH_3_ groups of the homopolymer PDMAM [27]. More specifically, the -CH_3_trans is shown at 3.1 ppm and the -CH_3_trans/cis is observed at 2.8–3 ppm [27]. This range is also associated with the -CH_2_- group (g) of epoxide ring, found at 2.6 and 2.8 ppm [28]. In the spectrum of the copolymer, the latter peak is overlapping with the peak attributed to the -CH- group (b) of PDMAM backbone. The -CH_2_- groups (a, c) of the copolymer backbone are identified in the range 2.1–1.2 ppm as multiple peaks. Specifically, the different stereochemical configurations of -CH- group of GMA are seen at 1.6–1.4 ppm (Hmmr_II,_ Hrmr_II_, Hmrm_I_, Hmrr_I_ and Hrr_I_, as shown in Figure S5). Finally, the resonance signals of the a-CH_3_ group (d) of GMA are split into three peaks appearing at 0.9 ppm (-CH_3_rr/rri), 1.1 ppm (-CH_3_rm/mrii) and 1.3 ppm (-CH_3_ mm/mmiii) due to different stereoisomerization [28,29,30]. Most of the aforementioned peaks of both structural units are detectable in the spectrum of the P(DMAΜ-co-GMA) copolymer. The chemical composition of the copolymer was determined from the integrals of the peaks b, g, h, f and e. It is found that the copolymer contains 33% mol/mol GMA units, in good agreement with the feed composition.

**Figure 3:**
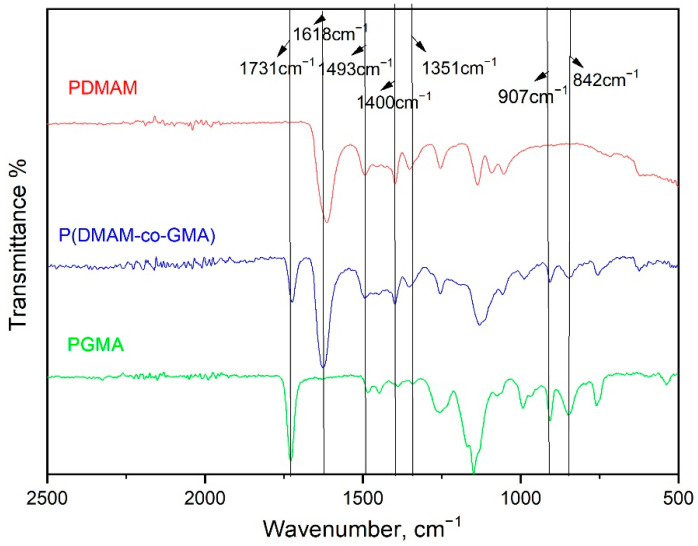
ATR-FTIR spectrum in the 500–2500 cm^−1^ region of the copolymer P(DMAM-co-GMA) in comparison with the spectra of the homopolymers PDMAM and PGMA.

The ATR-FTIR spectrum of P(DMAM-co-GMA) copolymer in the 500–2500 cm^−^^1^ region is presented in Figure 3, together with the spectra of the two homopolymers PDMAM and PGMA. The characteristic peak of the -N-H group of DMAM appears as a broad peak at ~3469 cm^−^^1^, while the peak at 2926 cm^−^^1^ is attributed to the -CH group of both DMAM and GMA units [31] (Figure S6). The carbonyl stretching of the ester group (-C=O) of GMA unit is observed in Figure 3 at 1731 cm^−^^1^ [28,31] and of DMAM unit at 1618 cm^−^^1^ [31]. The bending vibrations of methyl group are shown at 1493 cm^−^^1^ and 1400 cm^−^^1^ for both homopolymers. The -C-N group of DMAM unit is observed at 1351 cm^−^^1^ and the bending vibrations of epoxide ring of GMA unit are shown at 907 cm^−^^1^ and 842 cm^−^^1^. All characteristic peaks of GMA and DMAM units are detectable at the spectrum of the copolymer P(DMAM-co-GMA). As expected, the intensity of the peaks attributed to GMA units is low as a consequence of the rather low GMA content of the copolymer.

Through adequate mixing of aqueous solution of P(DMAΜ-co-GMA) and chitosan hydrochloride (Ch-NH_3_^+^Cl^−^), followed by casting at room temperature and further thermal treatment at 60 °C or 120 °C, flexible and transparent membranes (slightly brown when thermal treatment takes place at 120 °C) are obtained. Under these slightly acidic conditions, amine groups act as nucleophilic reagents and possibly attack the epoxide ring at the most substituted carbon (Figure 1b) [32].

**Figure 4:**
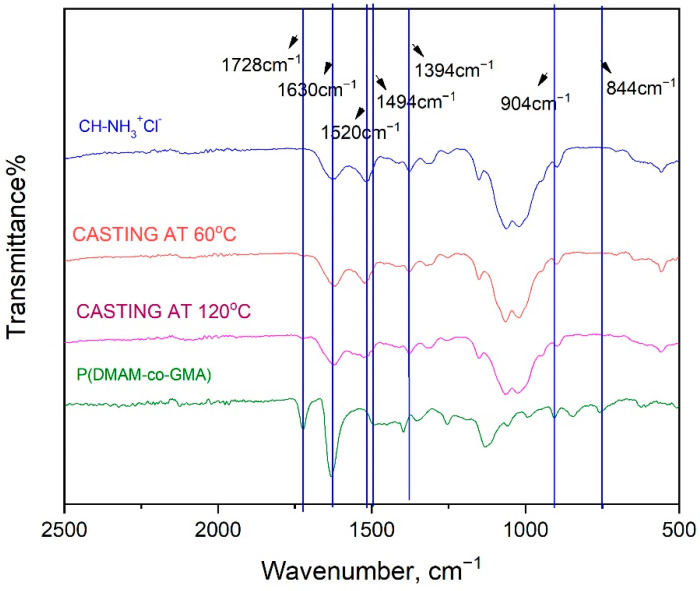
ATR-FTIR spectra in the 500–2500 cm^−^^1^ region of P(DMAM-co-GMA), Ch-NH_3_^+^Cl^−^ and the membrane Ch-NH_3_^+^Cl^−^/P(DMAM- GMA) 9:1 after thermal treatment at 60 °C and 120 °C.

The cross-linked chitosan-based membranes, after thermal treatment at 60 °C and 120 °C, were characterized through ATR-FTIR. As an example, the spectrum of the membrane Ch-NH_3_^+^Cl^−^/P(DMAM-GMA) 9:1 in the 500–2500 cm^−1^ region is shown in Figure 4. The functional groups of chitosan, -NH and -OH, including their intramolecular and intermolecular hydrogen bonding, appear at 3313 cm^−1^ (Figure S7). Furthermore, the stretching peak band at 2884 cm^−1^, associated with asymmetric bending vibration of -CH- group of chitosan, is also seen in Figure S7. The peak at 1630 cm^−1^ is associated with amide I (-C=O) of chitin, while amide II (-NH) and amide III (-C-N) are identified at 1520 cm^−1^ and 1376 cm^−1^, respectively, in the spectrum of chitosan hydrochloride. In addition, the peaks in the range 1190- 842 cm^−1^ correspond to stretching vibrations of -C-O-C- (glycidyl bond) of the polysaccharide backbone and the peak at 564 cm^−1^ is associated with -CH_3_ groups of the remaining acetyl group of chitin. The peak of copolymer P(DMAM-co-GMA) at 2894 cm^−1^ corresponds to the bending vibration of -CH group. The bending vibration at 1728 cm^−1^ and 1630 cm^−1^, which are characteristic of ester and amide groups of DMAM and GMA units, respectively, are also shown in the spectrum of P(DMAM-co-GMA) copolymer. Moreover, the bending vibrations of methyl group of GMA and DMAM units are observed at 1394 cm^−1^ and 1494 cm^−1^. All the aforementioned peaks are observed in both spectra of thermal-casted membranes Ch-NH_3_^+^Cl^−^/P(DMAM-GMA) 9:1. However, the peaks at 904 cm^−1^ and 844 cm^−1^, characteristic of epoxide ring of GMA, are now not observed, an indication that the thermal cross-linking between P(DMAM-co-GMA) copolymer and Ch-NH_3_^+^Cl^−^ takes place through the opening of the epoxide ring of GMA unit.

**Figure 5:**
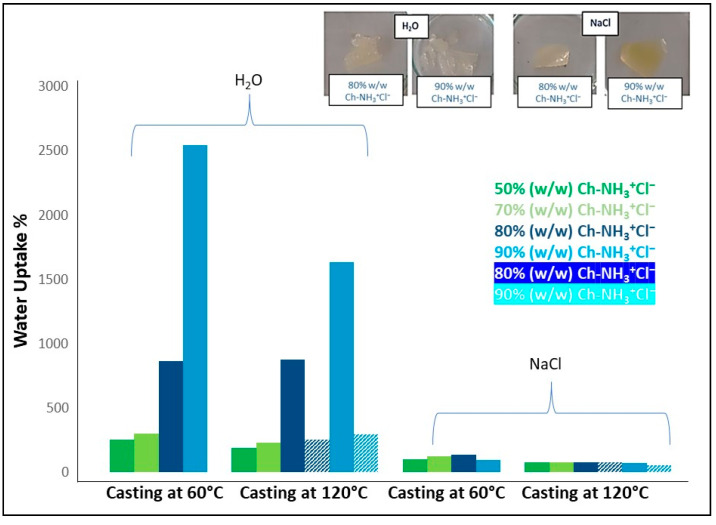
Water uptake of Ch-NH_3_^+^Cl^−^/P(DMAM-co-GMA) membranes with different molar ratio of Ch-NH_3_^+^Cl^−^, which were thermally treated at 60 °C and 120 °C. The appearance of the membranes with the highest Ch-NH_3_^+^Cl^−^ content after immersion in the solvents is shown in the inset.

The water uptake of the membranes in aqueous solutions depends strongly on the form of chitosan and the ionic strength. The results are shown in Figure 5. For the ionic Ch-NH_3_^+^Cl^−^/P(DMAM-co-GMA) membranes, the water uptake in pure water is high and increases substantially, reaching values of 2500% for the membrane with the higher chitosan content, as a consequence of the polyelectrolyte character of chitosan hydrochloride. Under these conditions, the membranes are highly swollen and smooth, as shown in the inset of Figure 5. In the case of immersion in aqueous 1 M NaCl solution, water uptake decreases significantly and values comparable to organic solvent uptake are determined, as a result of screening of charged groups of chitosan and the decrease of the internal osmotic pressure due to the addition of salt. The membranes now are just slightly swollen while retaining their flexibility (see inset of Figure 5). The membranes were also turned into the unprotonated uncharged form Ch-NH_2_/P(DMAM-co-GMA) through immersion in aqueous 1 M NaOH solutions (Figure S8). Due to the low water-solubility of Ch-NH_2_ and the absence of polyelectrolyte character of the membranes, water uptake in pure water is now very low. As a general remark, the water uptake of the membranes thermally treated at 120 °C is lower than that of those treated at 60 °C, as a consequence of the enhancement of the cross-linking reaction with temperature.

After the water uptake studies, all membranes were washed and dried, in order to determine the soluble content from the mass change according to Equation (2). The soluble content was limited (10–20%), due to the efficient thermal cross-linking between chitosan and P(DMAM-co-GMA) copolymer.

### 3.2. Adsorption Studies

**Figure 6:**
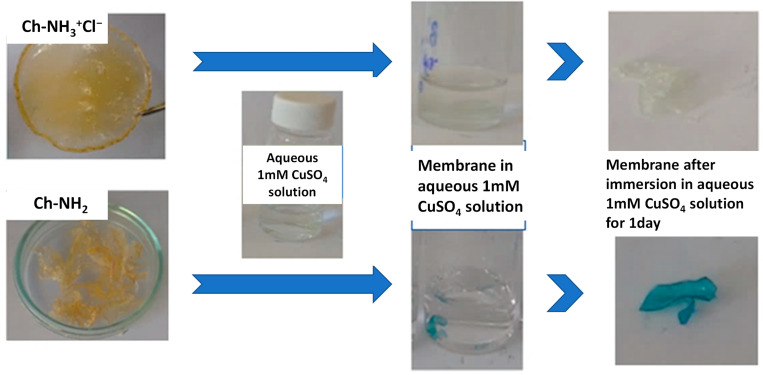
Representative images of the adsorption of copper ions by the membrane (Ch-NH_2_/P(DMAM-co-GMA) 8:2) compared to the appearance of the protonated Ch-NH_3_^+^Cl^−^/P(DMAM-co-GMA) 8:2 membrane, after immersion in aqueous 1 mM CuSO_4_ solution for 24 h.

The main goal of the present study was the potential application of the thus prepared cross-linked chitosan-based membranes as adsorbents of metal ions. To explore this possibility, Cu^2+^ ions were chosen as a typical example. As a first test, the Ch-NH_3_^+^Cl^−^/P(DMAM-co-GMA) 8:2 and Ch-NH_2_/P(DMAM-co-GMA) 8:2 membranes thermally treated at 120 °C were immersed into aqueous 1 mM CuSO_4_ solution for 24 h. Figure 6 shows the appearance of the membranes before and after this treatment. As seen, the protonated Ch-NH_3_^+^Cl^−^/P(DMAM-co-GMA) 8:2 membrane swells without any significant color change. In contrast, the unprotonated Ch-NH_2_/P(DMAM-co-GMA) 8:2 membrane turns strongly blue, indicating that the membrane adsorbs Cu^2+^ ions, apparently through complexation with the amine groups of Ch-NH_2._ A sample of a membrane before and after Cu^2+^ adsorption was characterized through SEM. As seen, the surface of the membrane is rather homogeneous, while the presence of copper is verified through EDS characterization (Figure S9).

**Figure 7:**
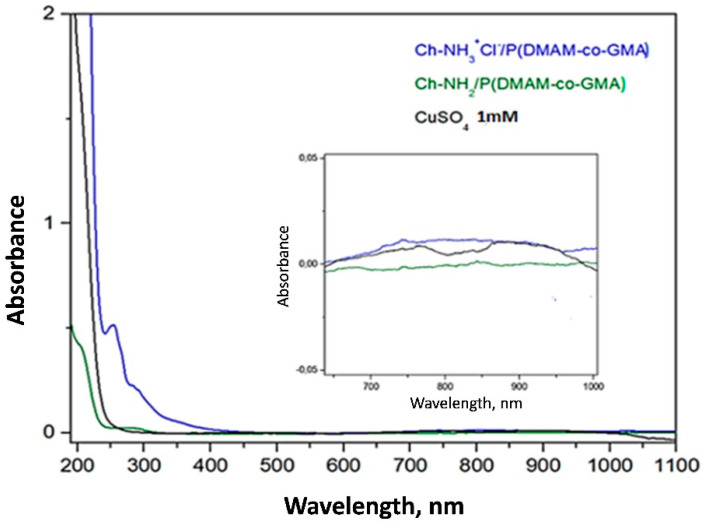
UV-vis spectrum of aqueous CuSO_4_ 1 mM solution before and after immersion of the membranes Ch-NH_3_^+^Cl^−^/P(DMAM-co-GMA_30_) 8:2 and Ch-NH_2_/P(DMAM-co-GMA) 8:2 for 24 h.

Taking into account these preliminary results, the UV-vis spectrum of the aqueous 1 mM CuSO_4_ solution, before and after the immersion of the membranes, was recorded (Figure 7). As the molarity of the aqueous CuSO_4_ solution is very low, the solutions are practically clear and the characteristic peak of d^9^ electrons of copper at ~800 nm is hardly detected. On the contrary, the other characteristic peak of copper ions at about 200 nm is strong and decreases substantially after the immersion of the unprotonated Ch-NH_2_/P(DMAM-co-GMA) 8:2 membrane. In the case of the protonated Ch-NH_3_^+^Cl^−^/P(DMAM-co-GMA) 8:2 membrane, on the other hand, the peak has higher values, while simultaneously new peaks appear at 255 nm, 270 nm and 283 nm. These absorption bands probably correspond to complexes of copper ions with Cl^−^ anions, namely [CuCl]^+^, [CuCl_2_] and [CuCl_3_]^−^ [33].

Having in mind the results for the unprotonated membrane, we checked the validity of the Beer–Lambert law at 200 nm. The UV-vis spectra recorded for aqueous CuSO_4_ solutions of known low concentrations are given in Figure S10. The variation of the absorbance at 200 nm with the molarity of Cu^2+^ ions is linear with a correlation coefficient (R^2^) of 0.9994 (Figure S11). To further verify the reliability of this detection methodology, samples of known concentration were characterized both through the aforementioned method and the method of bathocuproine. The results obtained from the two methods, as represented in Figure S12, practically coincide, especially for concentration lower than 0.3 mM, which is close to the upper limit of validity of the bathocuproine method [34].

#### 3.2.1. Adsorption Kinetics

**Figure 8:**
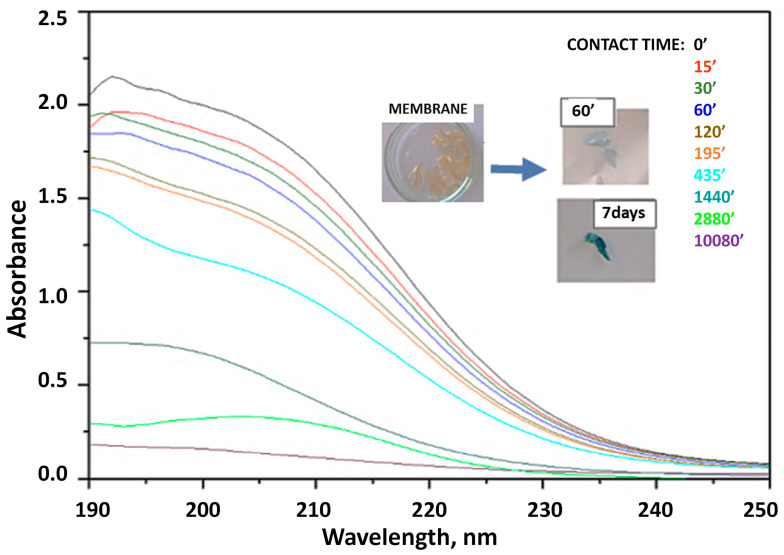
UV-vis spectra of aqueous CuSO_4_ 1.5 mM solution before and after immersion of the membrane Ch-NH_2_/P(DMAM-co-GMA) 9:1 with increasing contact time.

The kinetics of Cu^2+^ ion adsorption were studied using the Ch-NH_2_/P(DMAM-co-GMA) 9:1 membrane. The membrane was immersed in an aqueous 1.5 mM CuSO_4_ solution and the UV-vis spectra were recorded as a function of contact time (Figure 8). As seen, the absorption band at 200 nm decreases with contact time and eventually disappears, indicating the effective removal of Cu^2+^ ions from the aqueous solution. In fact, our results are encouraging, since Cu^2+^ concentration is decreased to the detection limits of this method, namely below ~0.1–0.2 mM (Figure S9). Moreover, adsorption can be visually detected, since the membrane turns gradually to blue. This color is observed already after a contact time of 60 min and gets deeper as the contact time increases (see inserted photos in Figure 8). Simultaneously, the membrane swells to reach a water uptake close to that observed in pure water for longer immersion periods (Figure S13).

To quantify the adsorption efficiency of Ch-NH_2_/P(DMAM-co-GMA) membranes the adsorption capacity (Q_t_, mg/g) was estimated as a function of contact time as:

(3) $$Q_{t}=\frac{\left(C_{0}-{\;C}_{t}\right)\;V}{m}$$

where C_0_ (mΜ) and C_t_ (mΜ) are the initial concentration and the concentration at time t (days) of Cu^2+^ ions, V (mL) is the volume of the solution and m (mg) is the initial mass of the membrane.

**Figure 9:**
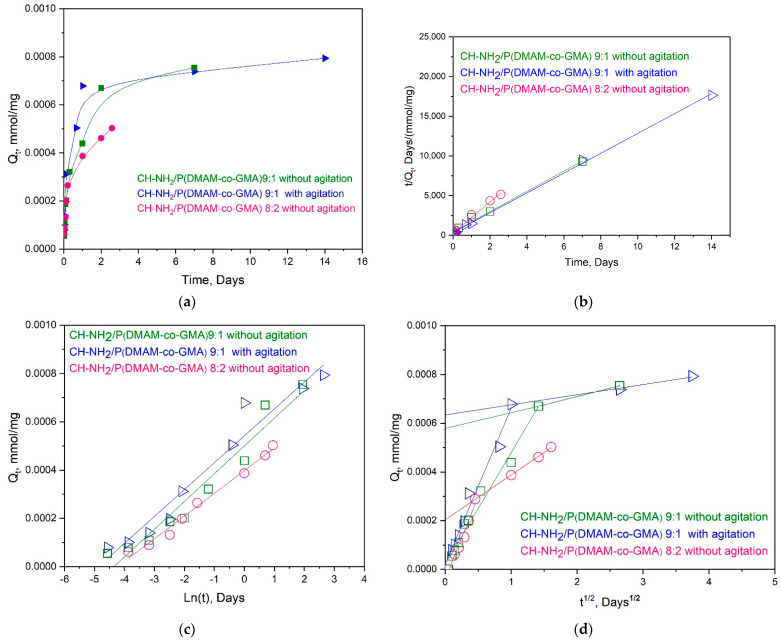
(**a**) Effect of contact time on adsorption capacity of the membranes Ch-NH_2_/P(DMAM-co-GMA) 9:1 and 8:2 in aqueous 1 mM CuSO_4_ solution, with or without magnetic stirring. Fitting of the experimental data with the (**b**) pseudo-second order, (**c**) Elovich and (**d**) Weber–Morris intraparticle diffusion model.

Initially, the influence of agitation conditions on adsorption was investigated. The dependence of Q_t_ on immersion time is depicted in Figure 9 for the Ch-NH_2_/P(DMAM-co-GMA) 9:1 membrane under conditions with and without magnetic stirring. As seen, the influence of magnetic stirring is rather marginal and just slightly higher Cu^2+^ ion removal is observed under stirring. For this reason, all other adsorption studies were performed without stirring. As a general observation in Figure 9, the adsorption capacity in all studies performed increases strongly within the first 1–2 days and tends to a plateau value for longer contact times. In addition, the influence of the membrane composition is also presented in Figure 9, where the adsorption capacity of the membranes Ch-NH_2_/P(DMAM-co-GMA) 9:1 and Ch-NH_2_/P(DMAM-co-GMA) 8:2 without magnetic stirring is compared. As observed, the Q_t_ values obtained for the first membrane are substantially higher than those for the second one, apparently reflecting the lower chitosan content of the Ch-NH_2_/P(DMAM-co-GMA) 8:2 membrane.

The results of Figure 9a were fitted to the most usual kinetics models [35], namely the pseudo-second order model, the Elovich model and the Weber–Morris intraparticle diffusion model, in Figure 9b, 9c and 9d, respectively.

The pseudo-second order model is based on the hypothesis that the adsorption process follows a second-order chemisorption mechanism, and it is described by the equation:

(4) $$\frac{t}{Q_{t}}=\frac{1}{K_{i}{\;Q}_{e}^{2}}+\frac{t}{Q_{e}}$$

where Q_e_ (mmol/mg) is the amount of adsorbed metal ions at equilibrium and K_i_ (mg/(g·day)) is the pseudo second-order adsorption rate constant.

The Elovich model is used to describe the chemical adsorption on heterogeneous adsorbents and is described by the equation:

(5) $$Q_{t}=\frac{1}{b}\ln\left(a\;b\right)+\frac{1}{b}\mathrm{lnt}$$

where a (mg/(g·min)) is the initial sorption rate and b (g/mg) is a parameter correlated with the degree of surface coverage and activation energy of chemisorption.

The Weber–Morris intraparticle diffusion mechanism is suggested when the intraparticle diffusion is the slowest stage of chemisorption and it is described by the equation:

(6) $$Q_{t}={\;K}_{i}\;\sqrt{t}+c$$

where c (mmol/mg) is a constant related to the boundary layer thickness and K_i_ (mg/(g·min^0.5^)) represents the intraparticle diffusion rate constant. The curve of this model is usually divided in two parts: the first linear part represents the boundary layer diffusion, followed by another linear part which is associated with the intraparticle diffusion.

From Table 1, where the fitting results are summarized, it is seen that the pseudo-second order (Figure 9b) is the best-fitted for all studied membranes, whereas the results cannot be well fitted using the Elovich model (Figure 9c). It should be noted that the pseudo-second order kinetics is usually adopted for the adsorption of metal ions by chitosan-based materials (see [16,17,19,20,21,22] and references therein), suggesting that the rate-determining step is chemisorption involving valence forces through sharing or exchange of electrons between adsorbent and sorbate. In fact, from the first model (Figure 9a), a Q_e_ value about 8 × 10^−4^ mmol/g and 5.1 × 10^−4^ mmol/g is found for the Ch-NH_2_/P(DMAM-co-GMA) 9:1 and Ch-NH_2_/P(DMAM-co-GMA) 8:2 membranes, respectively.

Adsorption kinetics are also well described using the intraparticle diffusion model, enabling us to understand the nature of the diffusion process. All curves show an initial linear portion, attributed to intraparticle diffusion. The Ki values for this portion fit rather well with the literature, i.e., they are similar or somewhat higher to those reported for the adsorption of Cu^2+^ ions by chitosan beads [36] or chitosan–clinoptilolite composite [37], respectively. Moreover, in our systems, these initial linear portions practically pass through the origin, suggesting that intraparticle diffusion is the major rate-limiting step.

#### 3.2.2. Adsorption Isotherms

For the investigation of the adsorption isotherms, samples of Ch-NH_2_/P(DMAM-co-GMA) membranes (m = 10 mg) were immersed in a series of aqueous CuSO_4_ solutions (5 mL) with concentration 0.01–5 mM at room temperature. To ascertain that equilibrium adsorption conditions were achieved, the samples were let to adsorb Cu^2+^ ions for 2 days, according to the kinetic findings. Three Ch-NH_2_/P(DMAM-co-GMA) membranes with compositions 9:1, 8:2, and 5:5 were studied.

**Figure 10:**
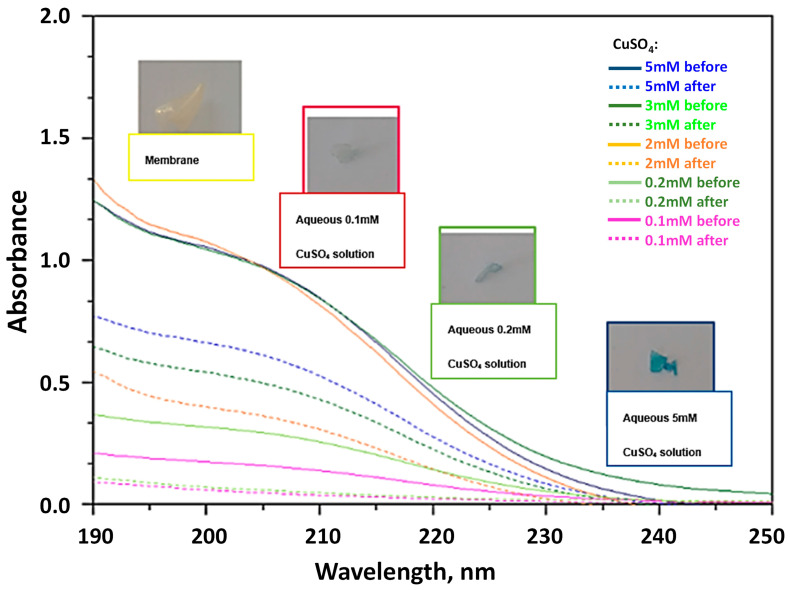
UV-vis spectra of a series of aqueous CuSO_4_ solutions, before (solid lines) and after (dotted lines) immersion of Ch-NH_2_/P(DMAM-co-GMA) 9:1 membranes. Characteristic images of the membranes during this study are inserted in the figure.

As an example, the UV-vis spectra of the solutions, before and after adsorption of Cu^2+^ ions by the Ch-NH_2_/P(DMAM-co-GMA) 9:1 membrane, are shown in Figure 10. It should be mentioned that for the most concentrated solutions (C_0_ > 1 mM), all other solutions were diluted to a final concentration C_0_ = 1 mM, in order to be within the validity limits of the calibration curve (Figure S11). In these cases, the solutions after adsorptions were respectively diluted, in order to be directly comparable to the mother solutions. From the UV-vis spectra of aqueous CuSO_4_ solutions, before and after immersion, it is evidenced that the adsorption of Cu^2+^ ions is highly efficient. Moreover, the binding of copper ions on active sites of Ch-NH_2_/P(DMAM-co-GMA) 9:1 membrane is visually detectable, already for C_0_ = 0.2 mM, from the appearance of the characteristic blue, which becomes more intense as the concentration of the solution increases.

The equilibrium adsorption capacity (Q_e_, mg/g) is determined as:

(7) $$Q_{e}=\frac{\left(C_{0}-{\;C}_{e}\right)\;V}{m}$$

where C_0_ (mΜ) and C_e_ (mΜ) are the initial and equilibrium concentration of Cu^2+^ ions, as found from the absorbance at 200 nm of the UV-vis spectrum or the bathocuproine method.

**Figure 11:**
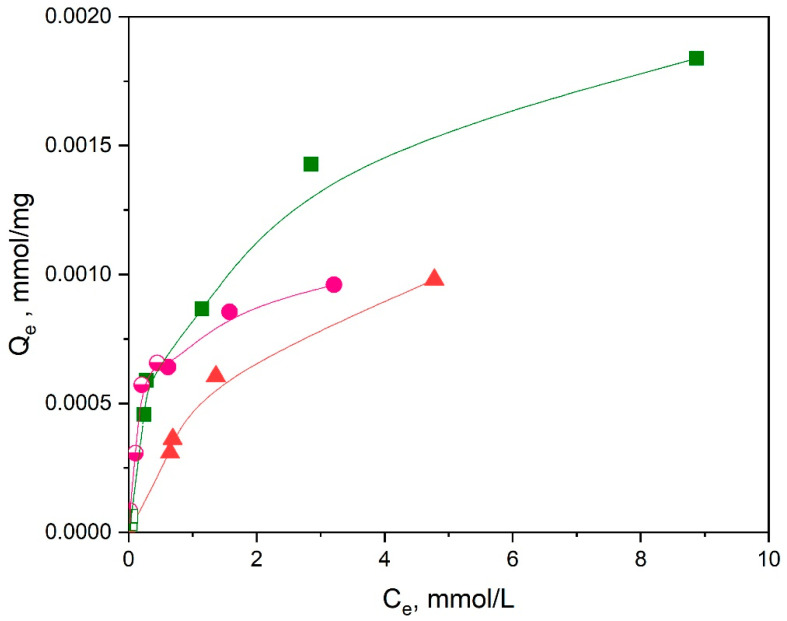
Adsorption isotherms of Cu^2+^ ions for membranes Ch-NH_2_/P(DMAM-co-GMA) 9:1 (green squares), 8:2 (magenta circles) and 5:5 (orange triangles), immersed in aqueous CuSO_4_ solutions for 2 days, without magnetic stirring. The results derived using the bathocuproine method are shown as semifilled symbols.

**Figure 12:**
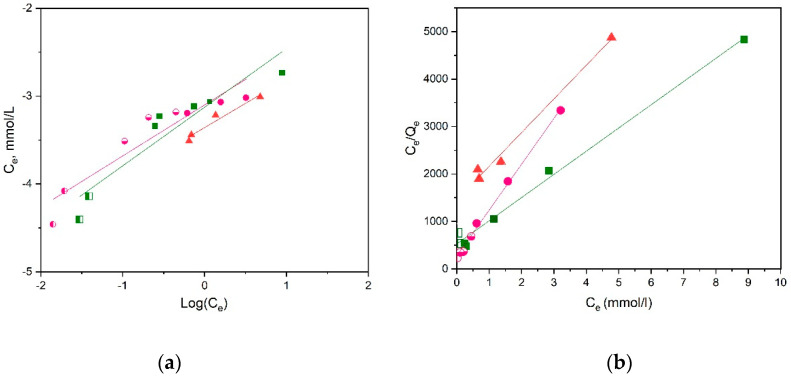
Freundlich (**a**) and Langmuir (**b**) isotherm models for the adsorption of Cu^2+^ for the studies reported in Figure 11.

The adsorption isotherms for the three membranes investigated are shown in Figure 11, while the fittings to linearized Langmuir and Freundlich models are presented in Figure 12. It is reminded that the linearized forms of these two models are, respectively:

(8) $$\frac{C_{e}}{Q_{e}}=\frac{1}{K_{L}{\;Q}_{m}}+\frac{C_{e}}{Q_{m}}$$

and

(9) $${\mathrm{logQ}}_{e}=\log K_{f}+\frac{1}{n}\log C_{e}$$

where Q_m_ (mg/g) represents the maximum adsorption capacity, K_L_ (L/mg) is the Langmuir constant, K_f_ (L/mg) is the Freundlich constant, and 1/n is related to the adsorption intensity [38].

As shown in Figure 12 and Table 2, the best-fitted model for the membranes seems to be the Langmuir model with R^2^ = 0.99, indicating that the functional groups of the material are uniformly occupied by the metal ions through a monolayer adsorption mechanism. Note that results obtained through different methods (adsorption at 200 nm and bathocuproine method) complement each other in these fittings. The determined Q_m_ values decrease as the chitosan content of the membrane decreases. In addition, the Q_m_ values are compared in Table 3 with respective values for Cu^2+^ ions adsorption of several chitosan-based materials. As seen, the results of the present study compare well with those of other cross-linked chitosan materials, indicating the potential of the present membranes for Cu^2+^ ion removal from aqueous solution for wastewater remediation processes.

#### 3.2.3. Regeneration and Reusability of Adsorbent

**Figure 13:**
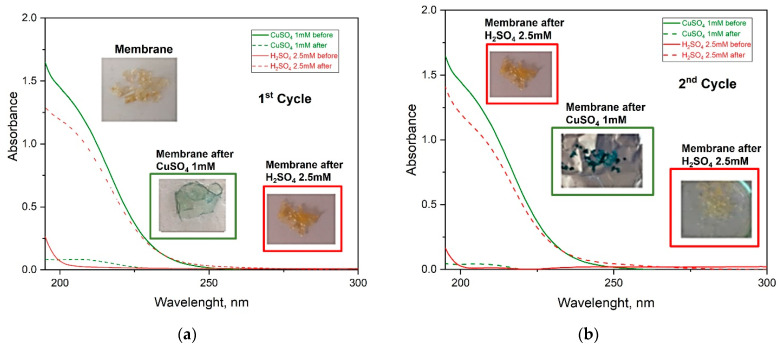

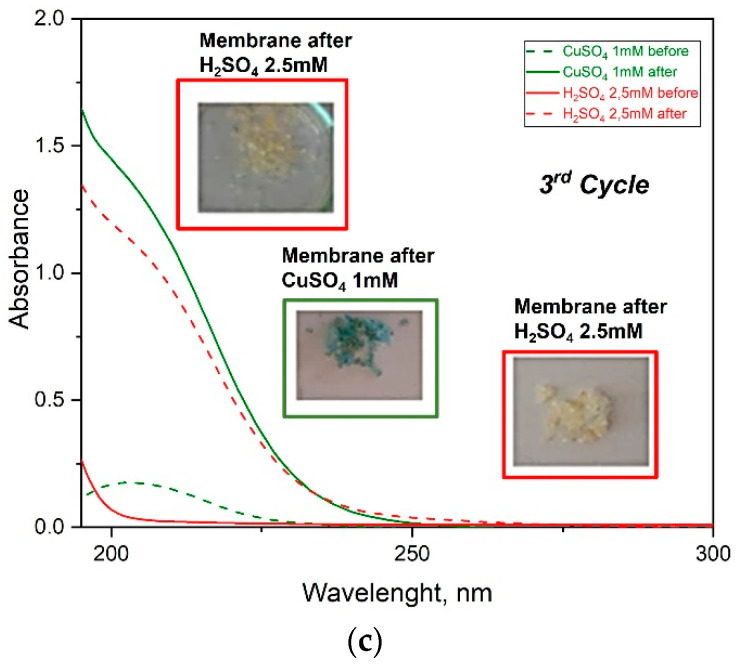
UV-vis spectra of adsorption–desorption studies of membrane Ch-NH_2_/P(DMAM-GMA) 8:2 for (**a**) first, (**b**) second and (**c**) third cycle.

To check the possibility of recovery of adsorbed Cu^2+^ ions and reusability of adsorbent, adsorption–desorption studies were performed. Usually, the desorption of metal ions and regeneration of different chitosan-based adsorbents take place by addition of acids (HCl), bases (NaOH) or chelating agents as ethylenediaminetetraacetic acid (EDTA) [60]. Here, the Ch-NH_2_/P(DMAM-GMA) 8:2 membrane was used for adsorption–desorption studies and an aqueous 2.5 mM H_2_SO_4_ solution was used as desorption agent. The performance of the membrane for three cycles was tested (Figure 13). After each cycle, the membrane was turned into the uncharged form through immersion in a NaOH solution, rinsing and drying. The pretreated membrane was initially immersed in an aqueous 1.2 mM CuSO_4_ solution, leading to an effective adsorption of Cu^2+^ ions, as verified by the blue color of the membrane and the substantial decrease of the adsorption band at 200 nm. The immersion of this membrane into the aqueous H_2_SO_4_ solution led to the successful desorption of copper ions, as confirmed by the decolorization of the membrane and through UV-vis spectroscopy of the solution.

**Figure 14:**
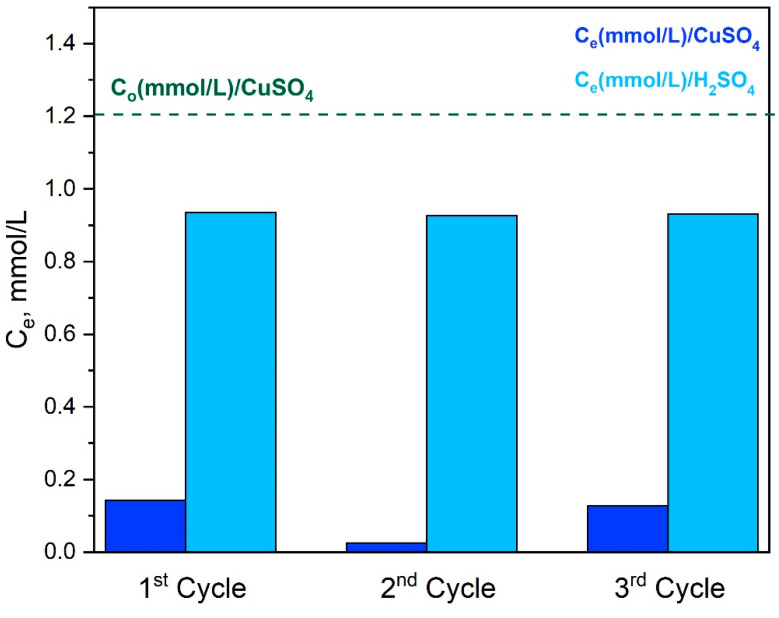
The values of C_e_ (mmol/L) during three cycles of Cu^2+^ ions adsorption–desorption study of Ch-NH_2_/P(DMAM-co-GMA) 8:2 membrane.

Since equal volumes of adsorption and desorption solution were used, the aforementioned results can be better quantified through the remaining equilibrium Cu^2+^ ion concentration, C_e_, after each step (Figure 14). Thus, as seen, the value of C_e_ after each adsorption step is roughly 0.2 mM, indicating that a quantity of Cu^2+^ ions ~1 mM had been practically quantitively desorbed after each cycle. This behavior is rather identical for three cycles, suggesting that the process is quite reproducible, and the synthesized membranes can be indeed regenerated, at least for some adsorption–desorption cycles.

## 4. Conclusions

In this work, cross-linked chitosan-based membranes were successfully synthesized by taking advantage of the reaction of amine groups of chitosan with the epoxide group of GMA during casting of aqueous chitosan hydrochloride/P(DMAM-co-GMA) solutions and subsequent thermal treatment. The protonated membranes were highly swollen in water, in contrast to the low swelling exhibited by the unprotonated membranes. The unprotonated membranes are effective adsorbents of Cu^2+^ ions, as confirmed by UV-vis spectroscopy of the adsorbate solutions. In fact, the kinetics of adsorption seems well adapted to the pseudo-second order and the intraparticle adsorption isotherms. The maximum adsorption capacity of the membranes is quite high (Q_m_ = 66–130 mg/g) and roughly decreases with the decrease of chitosan content. In fact, the Q_m_ values obtained are well comparable with those of other chitosan-based adsorbents. Moreover, it is shown that these materials can decrease the concentration of Cu^2+^ ions in water to a few ppm, while they can act as simple visual sensors for the detection of Cu^2+^ ions at concentration of about 0.2 mM.

Finally, it is shown that the membranes can successfully be regenerated in acidic solutions and reused for the adsorption of Cu^2+^ ions from water, at least for three cycles. Overall, the adsorption characteristics of the developed membranes (integrity, high Q_m_, easy regeneration) make these functional materials promising candidates for visual Cu^2+^ detection or effective removal of metal ions for water remediation applications.

## Figures and Tables

**Table 1:** Kinetic parameters for pseudo-second order, Elovich and intraparticle diffusion (Weber–Morris) models for membranes Ch-NH_2_/P(DMAM-co-GMA) 9:1 and Ch-NH_2_/P(DMAM-co-GMA) 8:2, with or without magnetic stirring.

Kinetic Models	Kinetic Parameters of Membranes Ch-NH_2_/P(DMAM-co-GMA)		
	9:1 with Magnetic Stirring	9:1 without Magnetic Stirring	8:2 without Magnetic Stirring
**Pseudo-second order** Q_e_ (mmol/mg)	Q_e_ = 8.01 × 10^−4^ R^2^ = 0.999	Q_e_ = 7.84 × 10^−4^ R^2^ = 0.993	Q_e_ = 5.11 × 10^−4^ R^2^ = 0.98
**Elovich** a (mg/(g·day)) b (g/mg)	a = 144.7 × 10^−4^ b = 8980 R^2^ = 0.96	a = 89.13 × 10^−4^ b = 8693 R^2^ = 0.95	a = 0.643 × 10^−4^ b = 10,624 R^2^ = 0.99
**Intraparticle Diffusion** **(Weber & Morris)** K_i1_, K_i2_ (mg/(g·min^0.5^))	K_i1_ = 0.64 K_i2_ = 4.2 × 10^−2^ R_1_^2^ = 0.98 R_2_^2^ = 0.996	K_i1_ = 0.69 K_i2_ = 4.6 × 10^−2^ R_1_^2^ = 0.98 R_2_^2^ = 0.98	K_i1_ = 0.68 K_i2_ = 0.18 R_1_^2^ = 0.96 R_2_^2^ = 0.9996

**Table 2:** Parameters for Langmuir and Freundlich models for the adsorption of Cu^2+^ ions by the Ch-NH_2_/P(DMAM-co-GMA) membranes with different contents of chitosan hydrochloride.

Isothermal Model	Composition of Membranes		
	9:1	5:5	8:2
**Langmuir** K_L_ (L/mg), Q_m_ (mg/g)	Κ_L_ = 1.1 Q_m_ = 130 R^2^ = 0.99	Κ_L_ = 3.5 Q_m_ = 66 R^2^ = 0.99	K_L_ = 0.5 Q_m_ = 90 R^2^ = 0.94
**Freundlich** K_f_ (L/mg)	K_f_ = 7.5 × 10^−4^ n = 1.5 R^2^ = 0.89	K_f_ = 7.9 × 10^−4^ N = 1.7 R^2^ = 0.88	K_f_ = 4.9 × 10^−4^ N = 1.8 R^2^ = 0.95

**Table 3:** Comparison of the maximum adsorption capacity based on Langmuir model of this study with other chitosan-based adsorbents.

Chitosan-Based Adsorbents	Q_m_ (mg/g)	Reference
Chitosan/cotton fiber	25	[39]
Chitosan/cellulose	53.2, 26.5	[40,41]
Chitosan or cross-linked chitosan	46–81, 35.5	[42,43]
Xanthate-modified magnetic chitosan	34.5	[44]
Chitosan/thiourea	66.7	[36]
Chitosan/alginate	67.7	[45]
Composite chitosan-based nanofibrous mats	79	[46]
Chitosan/ceramic alumina	86	[47]
Chitosan/activated carbon	90.9	[48]
Chitosan/glutaraldehyde microcapsules	100	[49]
Chitosan/EDTA	135	[50]
Carboxymethyl chitosan/graphene oxide	146	[51]
Chitosan-coated perlite beads	156	[52]
Chitosan/poly(acrylic acid) cross-linked	163	[53]
Chitosan/maleic anhydride	166	[54]
Chitosan/4-aminobenzoic acid	183	[55]
Magnetic chitosan/activated carbon composite	216.6	[56]
Chitosan/sulfydryl-functionalized graphene oxide	425	[57]
Cross-linked chitosan/waste active sludge char	490	[58]
Chitosan/linoptilolite	574, 719	[37,59]
Chitosan/P(MAM-co-GMA) membranes	66–130	This study

## Data Availability

The data presented in this study are available from the corresponding author upon reasonable request.

## Supplementary Materials

The following supporting information can be downloaded at https://www.mdpi.com/article/10.3390/ma16051926/s1. Figure S1. The chemical structure of chitosan. Figure S2. Schematic representation of intramolecular and intermolecular chelation of Cu^2+^ ions with chitosan units. Figure S3. Preparation of chitosan hydrochloride, Ch-NH_3_^+^Cl^−^. Figure S4. 1H ΝΜR spectrum of chitosan hydrochloride, Ch-NH_3_^+^Cl^−^. Figure S5. Schematic representation of stereochemical configurations of GMA units in PGMA, with respect to -H (left) και a-CH_3_ (right), along the methacrylate polymer chain. (The more shielded -H or a-CH_3_ is symbolized by “i” and the less shielded ones by “iii”). Figure S6. ATR-FTIR spectrum in the 2000–4000 cm^−1^ region of the copolymer P(DMAM-co-GMA), in comparison with the spectra of the homopolymers PDMAM and PGMA. Figure S7. ATR-FTIR spectra in the 2000–4000 cm^−1^ region of P(DMAM-co-GMA), ChNH_3_^+^Cl^−^ and the membrane Ch-NH_3_^+^Cl^−^/P(DMAM- GMA) 9:1 after thermal treatment at 60 °C and 120 °C. Figure S8. Schematic representation of deprotonation of Ch-NH3^+^Cl^−^/P(DMAM-co-GMA) membranes after immersion in aqueous 0.1 M NaOH solution for 24 h at room temperature. The appearance of the Ch-NH3^+^Cl^−^/P(DMAM-co-GMA) 8:2 membrane is shown, as example. Figure S9. Surface morphology and EDS characterization of the Ch-NH_2_/P(DMAM-co-GMA) 7:3 membrane (a) after preparation and thermal crosslinking at 120 °C (scale bar: 20 μm), and (b) after adsorption of Cu^2+^ ions (scale bar: 30 μm). Figure S10. UV-Vis spectra of aqueous CuSO_4_ solutions in the concentration range 0.25–1.5 mM. Figure S11. Calibration curve of the absorbance of aqueous CuSO_4_ solutions at 200 nm. Figure S12. Comparative results of measured and nominal concentrations of aqueous CuSO_4_ solutions, using the Bathocuproine method and the method of absorbance at 200 nm. Figure S13. Water Uptake% of Ch-NH_2_/P(DMAM-co-GMA) 8:2 and 9:1 membranes as a function of immersion time in aqueous 1.5 mM CuSO_4_.
