# Supplementary material for: Investigation of Cross-Linked Chitosan-Based Membranes as Potential Adsorbents for the Removal of Cu2+ Ions from Aqueous Solutions
Source: Materials (Basel). 2023 Feb 25;16(5):1926. doi: 10.3390/ma16051926 (PMC10004399; doi:10.3390/ma16051926)
Supplement: Supplementary file 1 [file materials-16-01926-s001.zip › materials-2224144-supplementary.pdf]

Supplementary Material

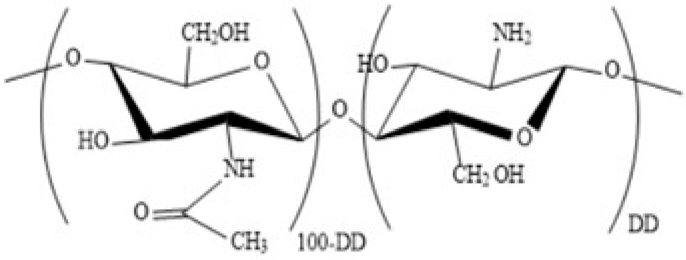

Figure S1. The chemical structure of chitosan.

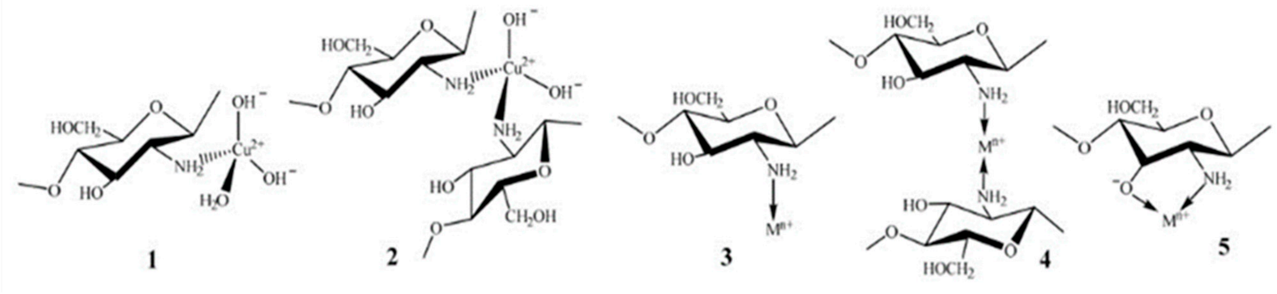

Figure S2. Schematic representation of intramolecular and intermolecular chelation of Cu^{2+} ions with chitosan units.

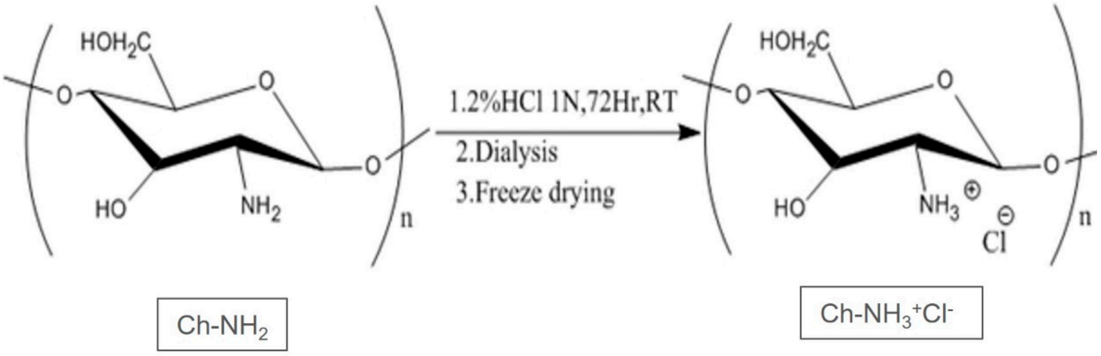

Figure S3. Preparation of chitosan hydrochloride, Ch-$NH_3^+Cl^-$.

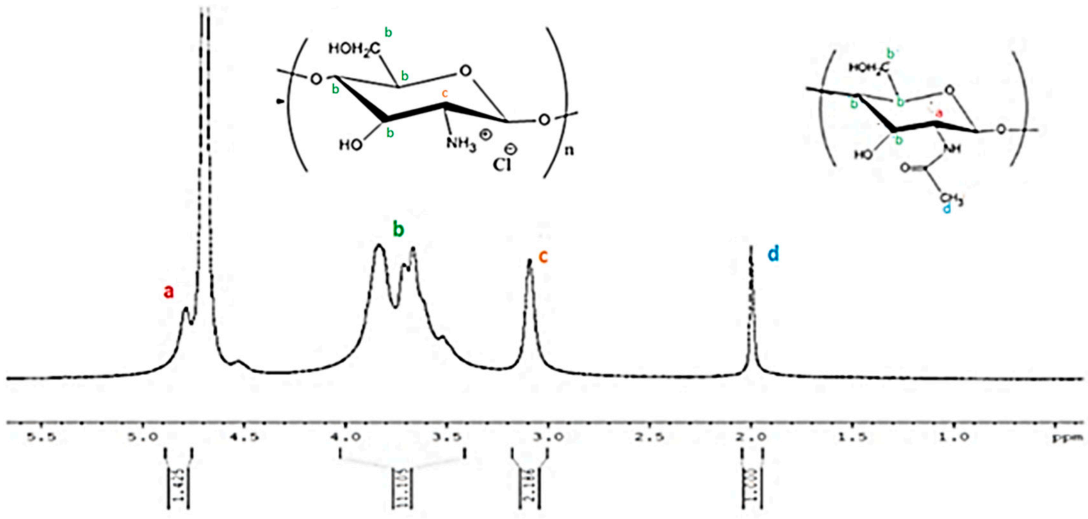

Figure S4. ^{1}H NMR spectrum of chitosan hydrochloride, Ch-$NH_3^+Cl^-$.

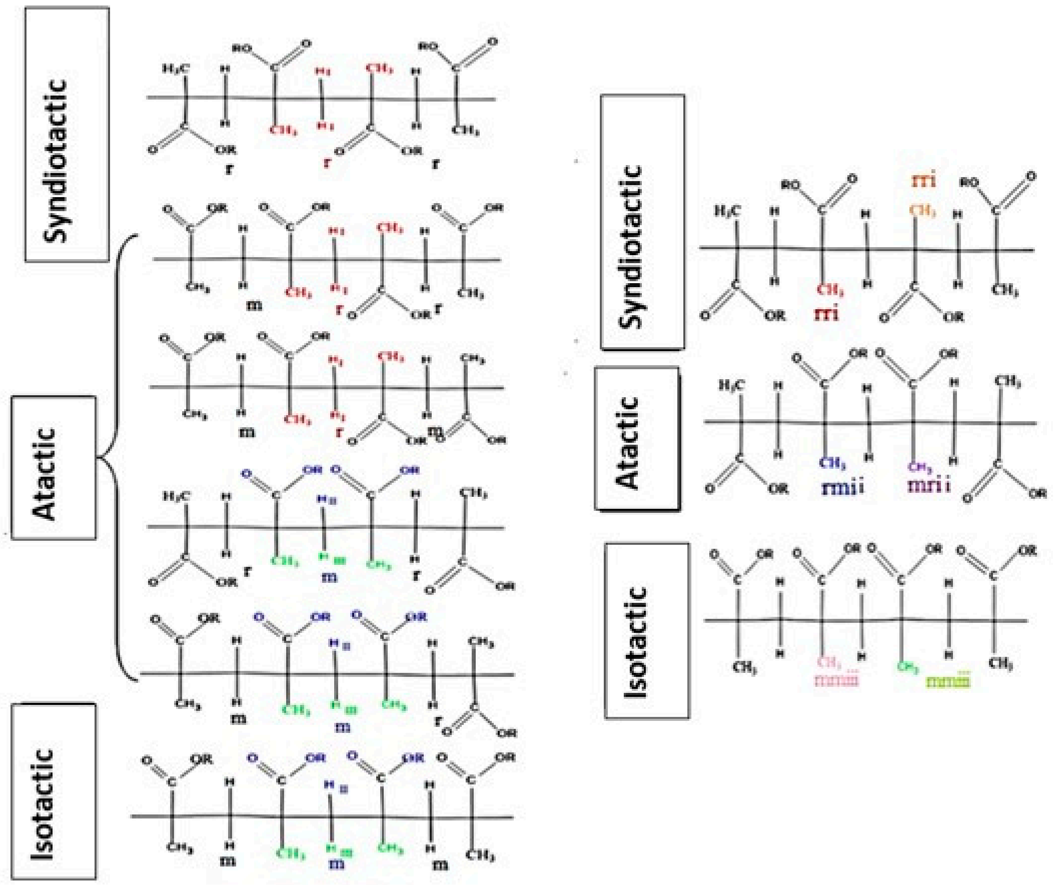

Figure S5. Schematic representation of stereochemical configurations of GMA units in PGMA, with respect to -H (left) και a-CH_3 (right), along the methacrylate polymer chain. (The more shielded -H or a-CH_3 is symbolized by ''i'' and the less shielded ones by ''iii'').

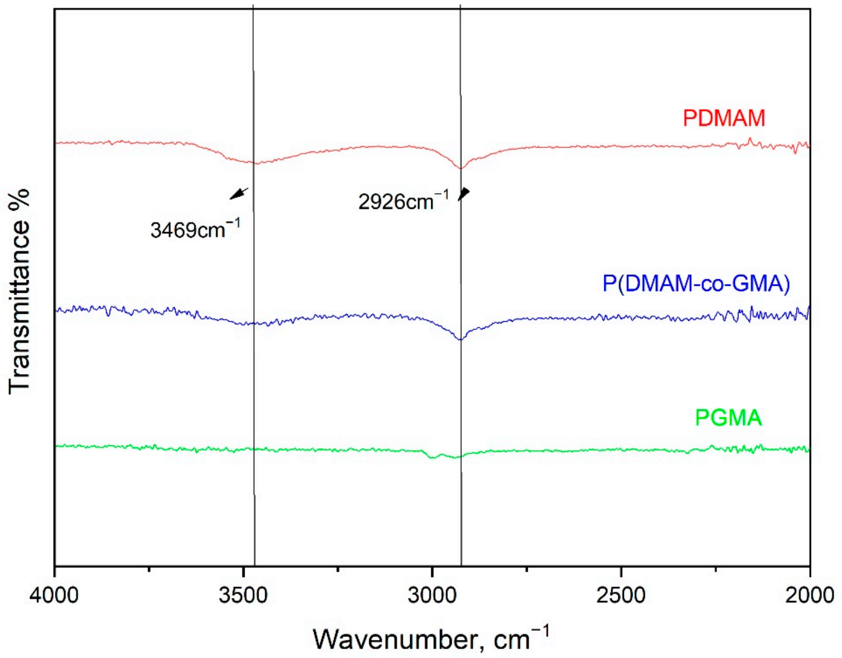

Figure S6. ATR-FTIR spectrum in the 2000-4000 cm^{-1} region of the copolymer P(DMAM-co-GMA), in comparison with the spectra of the homopolymers PDMAM and PGMA.

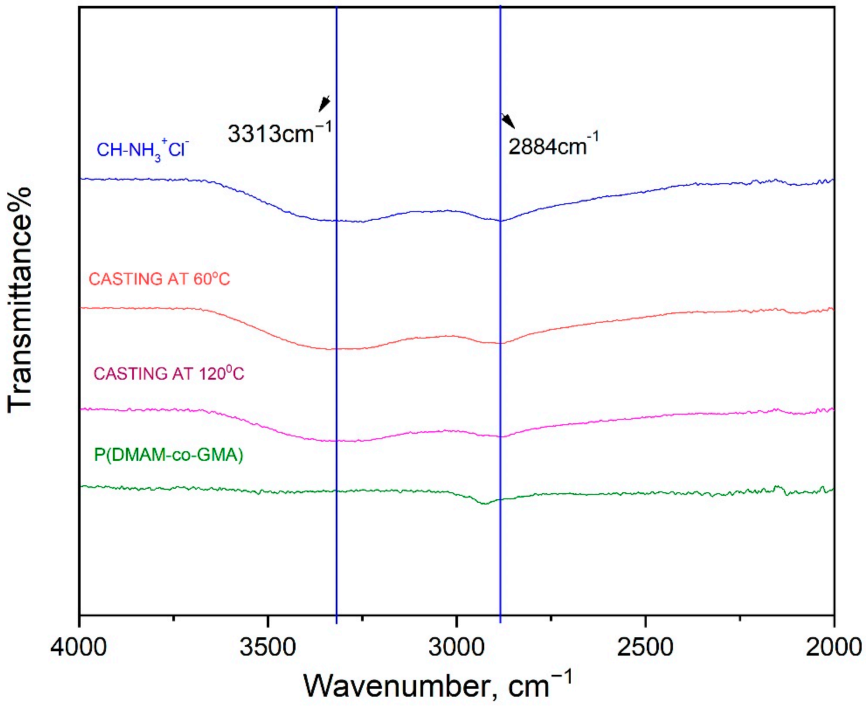

Figure S7. ATR-FTIR spectra in the 2000-4000 cm^{-1} region of P(DMAM-co-GMA), Ch$NH_3^+Cl^-$ and the membrane Ch-$NH_3^+Cl^-$ /P(DMAM- GMA) 9:1 after thermal treatment at 60°C and 120°C.

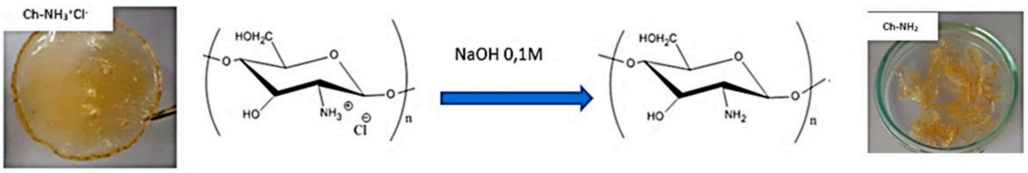

Figure S8. Schematic representation of deprotonation of Ch-$NH_3^+Cl^-$/P(DMAM-co-GMA) membranes after immersion in aqueous 0.1M NaOH solution for 24 hours at room temperature. The appearance of the Ch-$NH_3^+Cl^-$/P(DMAM-co-GMA) 8:2 membrane is shown, as example.

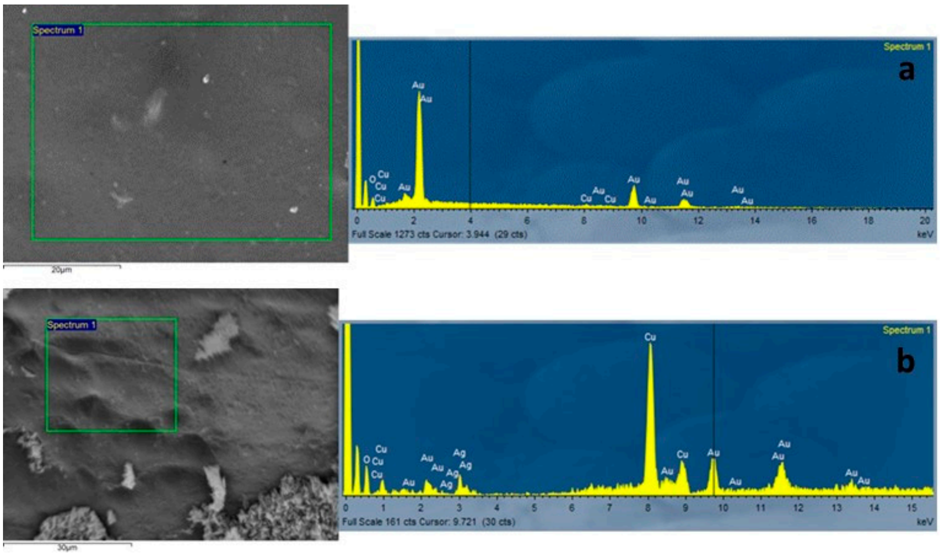

Figure S9. Surface morphology and EDS characterization of the Ch-NH_2/P(DMAM-co-GMA) 7:3 membrane (a) after preparation and thermal crosslinking at 120 °C (scale bar: 20 µm), and (b) after adsorption of Cu^{2+} ions (scale bar: 30 µm).

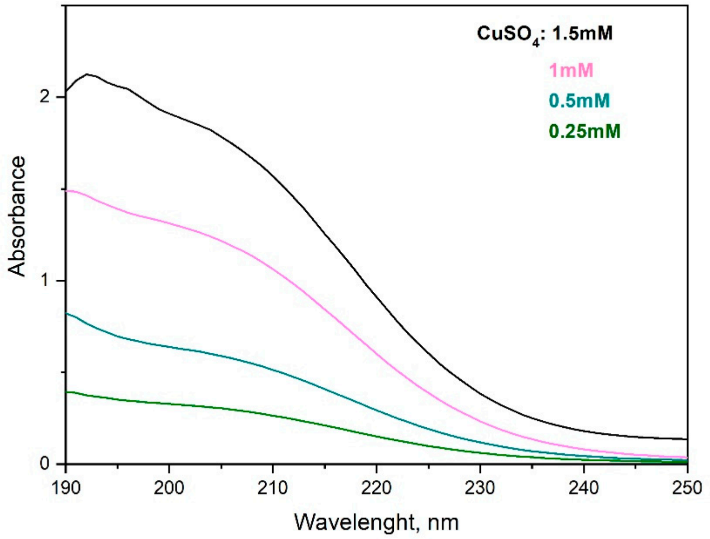

Figure S10. UV-Vis spectra of aqueous $CuSO_4$ solutions in the concentration range 0.25-1.5 mM.

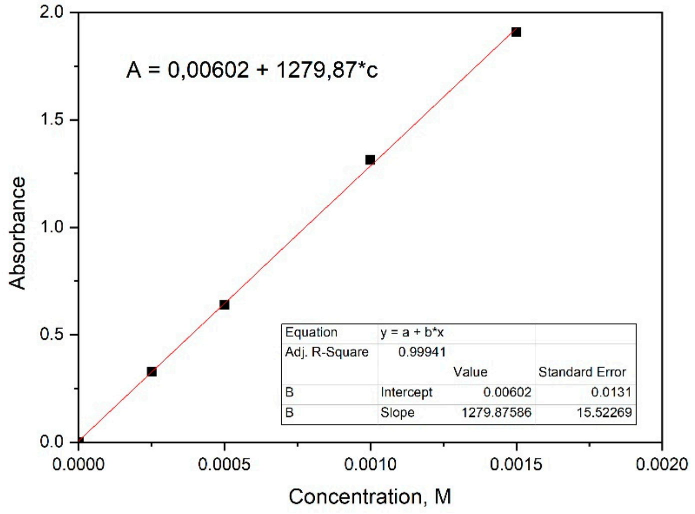

Figure S11. Calibration curve of the absorbance of aqueous $CuSO_4$ solutions at 200nm.

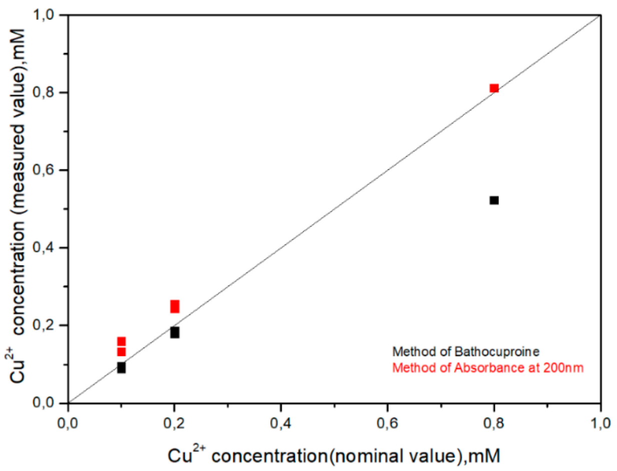

Figure S12. Comparative results of measured and nominal concentrations of aqueous $CuSO_4$ solutions, using the Bathocuproine method and the method of absorbance at 200nm.

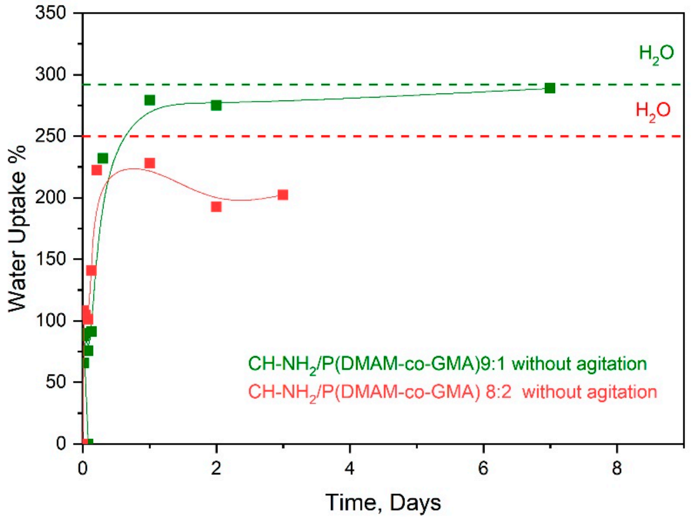

Figure S13. Water Uptake% of Ch-NH2/P(DMAM-co-GMA) 8:2 and 9:1 membranes as a function of immersion time in aqueous 1.5 mM $CuSO_4$.
